# Quality of life measurements as an indicator for timing of support after oesophagectomy for cancer: a prospective study

**DOI:** 10.1186/s12913-015-0747-x

**Published:** 2015-03-12

**Authors:** Marlene Malmström, Rosemarie Klefsgard, Bodil Ivarsson, Maria Roman, Jan Johansson

**Affiliations:** Clinical Sciences, Lund University, Lund, Sweden; Department of surgery, Skane University Hospital, Lund, Sweden; Department of cardio-thoracic surgery, Skane University Hospital, Lund, Sweden

**Keywords:** Impact factors, Oesophageal cancer, Oesophagectomy, Quality of life, Surgery, Timing, Supportive care

## Abstract

**Background:**

Oesophagectomy is a major procedure with known side effects and reduced postoperative quality of life (QOL). It has been shown that support of patients in their new life situation is often lacking. Knowledge about how QOL changes over time is fundamental for addressing patient needs and for determining the optimal timing of supportive care. The aim of this study was to identify QOL changes over time as well as factors that may impact patient QOL during the first year after oesophagectomy for cancer.

**Methods:**

Patients operated on for adenocarcinoma or squamous cell cancer of the oesophagus were included in this study. Seventy-nine patients completed the European Organisation for Research and Treatment of Cancer QOL questionnaires (QLQ-C30 and QLQ-OES18) before and 2, 4, 6, 9, and 12 months after surgery. A general linear model with repeated measurement analysis of variance was used for statistical testing.

**Results:**

There was a significant QOL nadir at 2 months compared to 12 months after surgery (QLQ-C30 function scales p < 0.001, symptom scales p < 0.001, QLQ-OES18 scales p < 0.001). Treatment with proton-pump inhibitors was associated with enhanced QOL according to QLQ-C30 symptom scales (p = 0.003) and OES-18 scales (p = 0.015), but age, gender and American Society of Anaesthesiologists classification did not significantly impact QOL.

**Conclusions:**

Patient QOL is severely hampered the first year after oesophagectomy for cancer, with a nadir at 2 months after surgery. Treatment with proton-pump inhibitors improved patient responses to symptom scales. Evidence of severely affected QOL after surgery indicates that these patients need support at an early stage after surgery. These results can be used by healthcare professionals to develop a postoperative supportive-care programme that is timed and better optimised to meet patient needs. Trial registration: EudraCT database 2009-009997-28.

## Background

Patients who have undergone oesophageal resections for cancer experience reduced quality of life (QOL) over a substantial period after surgery [1-6]. Patients face extensive changes in their daily life after surgery, including reduced physical and sometimes psychological capacities [7-12], problems that may be underestimated by healthcare providers. Several studies have focused on various perspectives of QOL [1-6] as well as on associations between e.g. QOL and disease characteristics [6]. However, there is a lack of knowledge about how patient QOL as a total concept fluctuates during the first postoperative year. This information is fundamental when aiming to develop a supportive-care programme that is adjusted and timed according to patient needs.

Oesophageal cancer is the eighth most common cancer worldwide [13]. After diagnosis with oesophageal cancer, the first treatment option for patients is surgery. Surgical resection with or without chemotherapy or chemoradiotherapy is the mainstay therapy for cure [14]. Oesophageal resections are major surgical procedures with long hospital stays and strenuous postoperative rehabilitation. Compared with other gastrointestinal surgical procedures, recovery for oesophageal resection is usually longer. Although survival after oesophageal cancer surgery has gradually improved, outcome in terms of survival depends mainly on tumour stage at the time of diagnosis [15]; the five-year survival rate remains only 31% [16].

Outcomes after surgery may be expressed in terms of survival rates, but also in terms of improvement in QOL. In order to distinguish between QOL in the broader sense and QOL connected with a patient’s health, the concept of health-related QOL is often used [17]. This parameter is a multidimensional construct that refers to the ‘subjective evaluation of one’s ability to perform usual tasks and their impact on one’s everyday physical, emotional, and social well-being’ [18]. The present study focuses on aspects of QOL connected with patient health; therefore, in this study, QOL refers to patient health-related QOL.

Symptoms associated with QOL in this patient group are often divided into general symptoms (e.g. fatigue, diarrhoea, appetite loss, and dyspnoea) and oesophageal-specific symptoms (e.g. eating problems, reflux, cough, and oesophageal pain) [1-4]. Nutrition problems (e.g. dysphagia, weight loss, lack of appetite, changed sense of taste, or dumping) [1,3,7,9-11,19-23] as well as problems with changed bowel habits [1,2,8,10,23] are widely discussed and are often highlighted as the dominating problems for patients after surgery.

Previous studies have identified different and sometimes contradictory factors that impact QOL. For example, age [1,21], sex [21], co-morbidity, and tumour stage [24] have been shown to affect QOL in some studies but not in others. In a study by Johansson et al. [25], proton-pump inhibitors (PPIs) were shown to exert a positive effect on anastomotic strictures, indicating that the effect of PPIs on QOL should be investigated.

To date, several studies have described the impact of oesophageal cancer surgery on QOL. However, those studies often focused on specific symptoms and functions and even if they provided important knowledge about patient life after surgery, they did not provide healthcare workers with a clear picture of when patient life is most severely affected. Knowledge about the timing of support is greatly needed in order to optimise patient support after surgery for oesophageal cancer.

### AIM

The aim of this study was to identify QOL changes over time as well as factors that may impact patient QOL during the first year after oesophagectomy for cancer.

## Methods

### Study design, setting, and sample

This descriptive prospective study was a separate part of a randomised controlled trial carried out at Skane University Hospital. The study was conducted with two separate aims: (1) to conduct repeated assessments of health-related QOL before and after surgery (addressed in the current study), and (2) randomised controlled evaluation of the effects of PPIs on postoperative anastomotic strictures [25]. There were no major differences in the distribution of patient demographics in the two randomised groups; therefore, in the current study we evaluated both groups together and adjusted the results according to the randomisation. Since this study was exploratory and lacked a control and a test group, no power calculation was needed.

Briefly, patients were eligible for inclusion in this study if they had undergone oesophageal resections with gastric-tube reconstruction due to oesophageal cancer in the distal oesophagus or at the gastro-oesophageal junction without major postoperative complications and with tumour-free resection margins (see list of inclusion and exclusion criteria’s). During data collection, 129 patients were available for inclusion. Forty-nine patients were excluded due to in-hospital death (n = 1), anastomotic leaks (n = 2), refusal to participate (n = 39), or other reasons (n = 7). After confirming the exclusion criteria, 80 patients were included in this study. One patient (n = 1) was excluded due to failure to attend follow-up. Demographic data are shown in Table 1.

**Table 1:** **Demographic characteristics of patients at baseline** (**N** = **79**)

	**N**	**%**
**Sex**		
Male	61	77.2
Female	18	22.8
**Age in years**		
Mean (standard deviation)	64.9 (8.7)	
Median	64.5	
Range	44.8-82.9	
**Age groups**		
44.8-61.9 years	26	32.9
62.0-68.6 years	27	34.2
69.5-82.9 years	26	33.9
ASA classification*		
1	13	16.5
2	40	50.6
3-4	20	25.3
Missing	6	7.6
**Result of randomisation**		
Control group (no treatment)	40	50.6
Intervention group (treatment)	39	49.4
**Anastomotic strictures**		
Yes	23	29.1
No	56	70.9

*American Society of Anaesthesiologists (ASA) physical status classification system: 1 = normal healthy patient; 2 = patient with mild systemic disease; 3 = patient with severe systemic disease; 4 = patient with severe systemic disease that is a constant threat to life.

### List of inclusion and exclusion criteria’s

### Inclusion criteria

- Patients with tumours of any stage in the distal third of the oesophagus, including type II tumours at the gastro-oesophageal junction.
- Transthoracic oesophageal resection with gastric-tube reconstructions and circular stapled anastomoses in the upper right chest.
- Postoperative clinical courses without complications and postoperative anastomotic radiograms without anastomotic leakage.
- Macro- and microscopically tumour-free upper resection margins.
- Willingness, physical and mental capability to comply with randomisation, and ability to follow the study protocol.
- Age >18 years
- Living in the south of Sweden (Skåne county)

### Exclusion criteria

- Preoperative or planned postoperative chemotherapy or radiotherapy to the tumour area known at the time of discharge from the hospital.
- Postoperative need for continuous treatment with PPIs or histamine-2 blockers or treatment with steroidal or non-steroidal anti-inflammatory drugs other than occasional use.
- Known allergy or side effects to PPIs preventing continuous treatment for one year
- Present drug or alcohol abuse
- Failure to attend at least one postoperative visit

Patient recruitment began two days before discharge from the hospital ward. All patients received both oral and written information about the study before providing informed consent. This study was conducted in accordance with the Declaration of Helsinki [26], was approved by the ethics committee at Lund University, Lund, Sweden (LU-693-02), and was registered in the EudraCT database (2009-009997-28) for clinical trials.

### Data collection

All patients were asked to complete QOL questionnaires before the operation and at 2, 4, 6, 9, and 12 months after surgery. Questionnaires were sent to the patients by mail and were sent back in an enclosed envelope. No reminders were sent out. Answer frequency appears in Table 2.

**Table 2:** **Answer frequency and drop outs over time** (**N** = **79**)

**Time of measurements**	**Answers**		**Deceased**	**Missing**	Answers possible*	
	**n**	**%**	**n**	**n**	**n**	**%**
Pre-operative	71	89.9	0	8		
2 months	64	81.0	0	15		
4 months	50	63.3	3	26	76	65.8
6 months	55	69.6	6	18	73	75.3
9 months	52	65.8	10	17	69	75.4
12 months	32	40.5	24	23	56	57.1

*Adjusted for deceased patients.

### Instruments and measurements

Data collection was based on European Organisation for Research and Treatment of Cancer (EORTC) validated questionnaires. The general QOL questionnaire QLQ-C30 (version 3.0) and the oesophagus-specific module QLQ-OES18 were used.

The EORTC QLQ-C30 questionnaire was developed to assess health-related QOL in patients with cancer. QLQ-C30 incorporates nine multi-item scales divided into five functional scales (physical, role, emotional, cognitive, and social), three symptom scales (fatigue, nausea/vomiting, and pain), and a global health and QOL scale. Several single-item symptom measures are also included in this instrument [27].

EORTC diagnosis-specific modules have been developed to cover specific diagnoses and to address symptoms and concerns specific to patient groups. The oesophagus-specific module QLQ-OES18 measures oesophageal cancer-specific symptoms and assesses four symptom scales (dysphagia, eating, reflux, and oesophageal pain) and six single items (trouble swallowing saliva, choking, dry mouth, taste, cough, and speech) [28]. Both QLQ-OES18 and QLQ-C30 are based on a Likert scale. Before analysis, patient responses were linearly transformed into a 0–100 scale and further processed according to the EORTC scoring manual [29]. High scores on symptom scales indicate more symptoms, while high scores on function scales indicate better function.

The American Society of Anaesthesiologists (ASA) score was collected from patient medical records and used in our statistical model as a potential impact factor. The ASA score is a classification of the patient’s physical status and ranges from 1 (‘normal healthy patient’) to six (‘brain-dead patient’) [30]. In our model, we controlled for ASA classifications 1 and 2 as separate variables and ASA classifications 3–4 as one variable.

Age was categorized into three groups.

### Statistical analysis

Results from the QLQ-C30 and QLQ-OES18 questionnaires were transformed into function scales and symptom scales according to instructions from the providers [27-29]. Imputation of missing values was done in two steps. Values missing from completed questionnaires were replaced according to the scoring manual of the instrument [29]. Missing values due to missing forms were replaced via mean-value imputation. We sought to evaluate general trends for the scales rather than to separately analyse each transformed scale. A priori, we established three separate statistical models: one for the set of function scales (QLQ-C30) and two separate sets for the symptom scales (QLQ-C30 and QLQ-OES18). Preoperative and postoperative assessments at 2, 4, 6, 9, and 12 months after surgery were included into each of the three statistical models. A general linear model with repeated measurement analysis of variance was used. Results from the first postoperative year were evaluated as a composite time parameter and adjusted for the following potential impact factors: gender, age, ASA score, randomisation of the study population (PPIs or no treatment), and whether an anastomotic stricture appeared after surgery. Contrasts were used to compare the results from each evaluated pre- or postoperative occasion with the results of the 12-month assessments. Interaction analyses were carried out between the composed time parameter and each of the factors gender, age, ASA score, randomisation, and anastomotic stricture. The original data did not follow a perfect normal distribution. However, repeated-measurements analysis of variance was used due to its robustness to deviations from the normal distribution and because non-parametric statistical tests with this complex set-up are not available. When Mauchly’s test of sphericity indicated deviations from sphericity, adjustments of the degrees of freedom were made according to the Huynh-Feldt correction. Residuals were graphically checked for constant variance, normality, independence, and linearity. P-values <0.05 were considered significant. Calculations were performed with the SPSS 18 package (Chicago, USA).

## Results

A total of 79 patients, mostly men (77.2%), were included in this study. The mean age was 64.9 years, with a standard deviation of 8.7 years (Table 1). The answer frequency was 89.9% at baseline compared with 57.1% at the 12-month follow-up (adjusted for deceased patients; Table 2).

**Figure 1:**
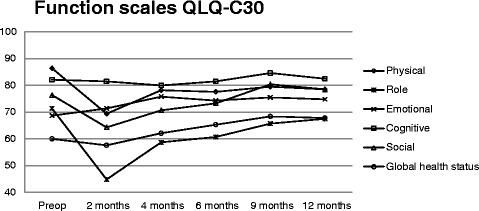
**Mean values of each QLQ-** **C30 function scale during the study year.** There was a significant deviation at 2 months compared to 12 months after surgery (p < 0.001). For function scales, a high score indicates a high level of functioning. For global health status, a high score indicates high QOL. Preop, preoperative.

Results for the function scales (QLQ-C30) exhibited an overall significant deviation in experienced function levels over time (p = 0.006). This change manifested as a significant peak of adverse functions at two months after surgery versus 12 months after surgery (p < 0.001; Figure 1). Results were not significantly influenced by gender (p = 0.379), age (p = 0.696), ASA score (p = 0.338), randomisation (p = 0.081), or whether an anastomotic stricture appeared after surgery (p = 0.732). Interaction analyses indicated no additional interactions among the evaluated variables during the study period.

**Figure 2:**
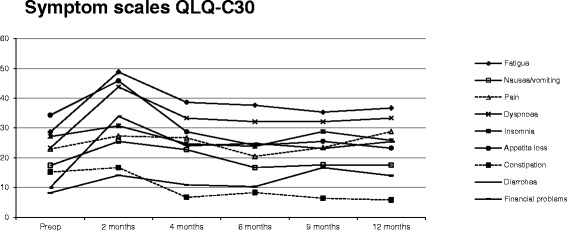
**Mean values of each QLQ-** **C30 symptom scale during the study year.** There was a significant deviation at 2 months compared to 12 months after surgery (p < 0.001). For symptom scales, a high score indicates more problems. Preop, pre-operative.

Results for the QLQ-C30 symptom scales also indicated an overall significant change in experienced symptoms over time (p < 0.001), with a significant peak of symptoms at two months after surgery compared to 12 months after surgery (p < 0.001; Figure 2). Overall, results were significantly impacted by the outcome of the randomisation (PPIs or no treatment; p = 0.003), but not by gender (p = 0.319), age (p = 0.696), ASA score (p = 0.338), or whether an anastomotic stricture appeared after surgery (p = 0.732). Interaction analyses indicated no additional interactions among the evaluated variables during the study period.

**Figure 3:**
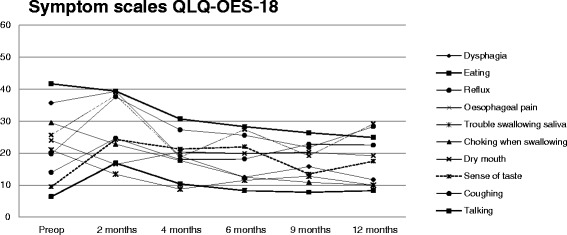
**Mean values for each QLQ**-**OES-** **18 symptom scale during the study year.** There was a significant change at 2 months compared to 12 months after surgery (p < 0.001). For symptom scales, a high score indicates more problems. Preop, pre-operative.

Results for the QLQ-OES18 symptom scales showed an overall significant change in symptoms over time (p < 0.001) with a significant peak of symptoms at two months after surgery compared to 12 months after surgery (p < 0.001; Figure 3). Results were significantly impacted by randomisation to PPIs or to no treatment (p = 0.015), but not by gender (p = 0.428), age (p = 0.812), ASA score (p = 0.900), or whether an anastomotic stricture appeared after surgery (p = 0.689). Interaction analyses indicated that patients with anastomotic strictures had more adverse symptoms at two months than at 12 months after surgery (p = 0.004). No other significant interactions were found.

## Discussion

### Methodological considerations

In this study, EORTC questionnaires were used to evaluate patient QOL, focusing on how symptoms and function fluctuate over the first postoperative year. Earlier studies reported that QOL is negatively affected after oesophagectomy within specific symptom and function areas [1-4,6], but no studies focused on when interventions are most needed. In order to focus on when patient QOL is most severely impaired during the first postoperative year, we performed a composite analysis of the components of the function and symptom scales instead of performing multiple analyses of each individual component of the scales. One potential limitation of this study is that it does not yield information about specific symptoms or functions. However, it provides a comprehensive picture of how QOL changes over time –information that is important when planning support interventions for these patients. Another potential limitation is that information about whether each patient lives alone was not available, which needs to be taken into account when interpreting our results.

This study of QOL was a separate part of a randomised controlled trial of the effect of PPIs on anastomotic strictures after oesophagetomy with gastric-tube reconstructions [25]. The fact that this study was a part of a trial in which patients were randomised to two arms (PPI treatment or no treatment) could be a source of potential bias. However, all patients selected for the study met the list of inclusion criteria irrespective of the study arm into which they were randomised. Since no major differences in the distributions of demographics were identified between the two arms (data not shown), all patients included in this study were evaluated together. Final findings were adjusted according to randomisation to PPI treatment or no treatment; patients who received PPIs had better outcomes than patients without PPIs. Hence, this study design enabled us to control for the potential impact of treatment on patient QOL, a strength of this study.

During data collection, approximately 30% of the included patients died, several due to relapse of their cancer. It is realistic to believe that those patients would have suffered from more symptoms than patients that did not suffer relapse. Although this scenario would probably have affected the answers of these patients, we did not adjust this study accordingly.

### Consideration of results

The results of this study indicated that patient QOL is negatively affected after oesophagectomy, with a nadir at two months compared to 12 months after surgery. These dynamics are likely due to several factors, such as persistent surgery-related ailments that exert both physical and psychological effects; further, patients were discharged from the ‘safe’ hospital setting, which included all necessary support, and struggled to adapt to a new life situation that included symptoms and ailments at home [5,8]. Earlier studies have shown a complex picture of patient QOL after oesophagectomy. Some studies reported that QOL was satisfactory or good [22,31,32], while others stated that it was reduced for a substantial period after surgery [1-3,23]. Several studies focused on differences in patient QOL at various time points after surgery [2,23,33]. In contrast to this study six response measurements during the first postoperative year, other studies often included long intervals between measurements points, with only a few measurement points during follow-up, or focused on specific symptoms and functions. Our intensive following of our patient group enabled us to draw more reliable conclusions about how QOL changes during the first postoperative year.

With good knowledge of underlying relevant factors that impact patient QOL, healthcare professionals can better tailor postoperative supportive-care programmes. This study demonstrated that treatment with PPIs, but not age, gender, or ASA classification, significantly impacted patient QOL (symptom scales). Gastro-oesophageal reflux is a prominent problem for this patient group [1,3,21]. For example, Lagergren et al. [3] reported that patients suffered from problems with reflux as long as 3 years after surgery. Taken together, these results suggest that treatment with PPIs has a positive impact on patient QOL and lowers the risk of oesophageal strictures after surgery [25]. Thus, PPIs could be recommended during the first postoperative year.

Earlier studies of patient experiences concluded that patients require support in handling persistent problems after surgery [34] and that postoperative support should address the patients’ physical, psychological, and social concerns [12,34]. The current study demonstrated that patient QOL is most severely reduced 2 months after surgery, which indicates that patients need support at an early stage. Supportive-care programmes within other cancer contexts exerted positive effects on QOL [35,36], reduced unmet supportive-care needs, and improved continuity of care [35]. However, earlier intervention studies that sought to support these patients after surgery reported divergent results on QOL [37-39]. Since our study showed that patient QOL is most severely hampered two months after surgery, the timing of support after surgery is essential. The current study suggests a great need for proactive supportive-care programmes for these patients; in hospital, patients should be prepared for life after surgery, and after discharge, physical and psychological support should be provided to patients in their new life situation. We therefore suggest enhancing patient QOL by combining a supportive-care programme (including a discharge meeting focusing on the post-surgery period) with nurse-led proactive telephone contacts that address individual needs during the first year after surgery. Further studies testing such a programme are needed.

## Conclusions

The current study demonstrates that patient QOL is severely reduced during the first year after oesophagectomy for cancer, with a nadir at 2 months after surgery. Treatment with PPIs improves patient responses on symptom scales. The severe effect on QOL after surgery indicates that these patients need support soon after surgery. These results can be used to help healthcare professionals develop postoperative supportive-care programmes that are better timed and optimised to patient needs.
